# Non-invasive Systemic Hemodynamic Index in Vascular Risk Stratification Tailored for Hypertensives

**DOI:** 10.3389/fcvm.2021.744349

**Published:** 2021-11-22

**Authors:** Jianning Zhang, Jiawen Liang, Xiaoyu Zhang, Chen Su, Jiang He, Yumin Qiu, Zhe Zhou, Zhichao Wang, Bing Dong, Qiang Tu, Shiyue Xu, Wenhao Xia, Jun Tao

**Affiliations:** ^1^Department of Hypertension and Vascular Disease, The First Affiliated Hospital of Sun Yat-sen University, Guangzhou, China; ^2^National-Guangdong Joint Engineering Laboratory for Diagnosis and Treatment of Vascular Disease, The First Affiliated Hospital of Sun Yat-sen University, Guangzhou, China; ^3^Key Laboratory on Assisted Circulation of Ministry of Health, The First Affiliated Hospital of Sun Yat-sen University, Guangzhou, China

**Keywords:** hypertension, vascular function, impedance cardiography (ICG), hemodynamics, surrogate biomarker

## Abstract

Vascular dysfunction is a key hallmark of hypertension and related cardiovascular outcomes. As a well-known hemodynamic disease, hypertension is characterized by abnormal ventricular-vascular interactions. Complementing non-invasive systemic hemodynamics in hypertensive vascular risk assessment is of promising significance. We aimed to investigate the effects of abnormal hemodynamic states other than elevated blood pressure on vascular damage and establish a united index of systemic hemodynamics for generalized vascular risk evaluation. Non-invasive systemic hemodynamics, assessed by impedance cardiography, was compared among blood pressure stages. Vascular function was evaluated by flow-mediated dilation (FMD) and brachial-ankle pulse wave velocity (baPWV). Systemic hemodynamics was obtained from a total of 88 enrollees with a mean (±SD) systolic blood pressure 140 (±17) mm Hg, and aged 17 to 91 years. Both stroke systemic vascular resistance index and left stroke work index exhibited a significant alteration among blood pressure stages (*p* < 0.001; *p* = 0.01, respectively), whereas heterogeneous hemodynamic and vascular function subsets existed within similar blood pressure. In addition, blood pressure categories failed to recognize between-group differences in endothelial dysfunction (*p* = 0.88) and arterial stiffness (*p* = 0.26). An increase in myocardial contractility and a parallel decrease in afterload was associated with the decline of vascular dysfunction. Systemic Hemodynamic Index (SHI), as a surrogate marker, demonstrated a significantly negative correlation with vascular damage index (VDI, *r* = −0.49, *p* < 0.001). These findings illustrate that systemic hemodynamics underlying hypertensives provides more vascular information. The SHI/VDI score may be a feasible tool for cardiovascular function assessment.

## Introduction

Approximately 1.13 billion people worldwide suffer from hypertension (1), which is the leading cause of premature death (2–4). Guidelines have urged for primary prevention in accordance with qualitative 10-year risk estimation, based on traditional atherosclerotic cardiovascular disease (ASCVD) risk factors in earlier blood pressure stages (5, 6). Elevated blood pressure, accompanied by other cardiovascular risk factors, contributes to vascular dysfunction (7, 8). Dual standardization of blood pressure and vascular function may be optimal for hypertension residual risk management. Large arterial stiffness has been viewed as a robust independent predictor of cardiovascular events and all-cause mortality in hypertensives (9, 10). Intensive blood pressure control is beneficial for attenuation of increases in arterial stiffness, which may account for reduced cardiovascular morbidity and mortality in the Systolic Blood Pressure Intervention Trial (SPRINT) trial (11, 12). Of note, our recent data pooled from a large-scale community-based Chinese cohort further reveal that arterial stiffness is associated with worse clinical outcomes than blood pressure alone (13). Moreover, endothelial dysfunction has become a potential target of a therapeutic approach for hypertension (14, 15). Therefore, several attempts (16–19) have been made to revise the Framingham risk score addressing the merits of brachial-ankle pulse wave velocity (baPWV), and/or brachial flow-mediated dilation (FMD), namely, vascular function. Hence, we propose that the concept of integrated vascular damage index (VDI) provides the framework to develop surrogate markers to identify hypertensives with varied vascular conditions.

Hemodynamics, reflecting ventricular-vascular structure and function interaction, is responsible for the pathophysiology of hypertension (20, 21). As an end-result of multivectorial, pulsatile modulations by volume, inotropy, and vasoactivity (22), it explains the substantially different hemodynamic phenotypes within identical blood pressure. Several non-invasive hemodynamic parameters measured by impedance cardiography (ICG), such as systemic vascular resistance and volume and arterial compliance, has been utilized in incident hypertension prediction (23), resistant hypertension management (24–26), or been recognized as predictors of 10-year all-cause mortality in hypertensives (27). Thus, compensation of hemodynamic indices in vascular risk stratification may optimize risk prediction tools for primary prevention of adverse cardiovascular outcomes. However, no researches have yet established a united systemic hemodynamics index (SHI) and investigated its role in vascular risk stratification.

In this study, overall non-invasive hemodynamic parameters, assessed by ICG technique, were presented to estimate alteration of vascular dysfunction. We aimed to construct synthetic surrogate markers, VDI and SHI, by principal component analysis (PCA)-based method, and explore their correlations in both controlled and uncontrolled hypertensives.

## Materials and Methods

### Study Population

In this retrospective, cross-sectional study, we recruited 88 enrollees, aged 17–91 years, who underwent an ICG test at the Department of Hypertension and Cardiovascular Disease, the First Affiliated Hospital of Sun Yat-sen University from March 2016 to December 2016. Participants with new-onset hypertension (0–5 years duration) and more than 5 years of history of hypertension were included. Among these participants, 46 were on antihypertensive treatments before enrollment. Patients who had secondary hypertension, untreated diabetes, acute infectious diseases, active malignant tumor, chronic renal failure, acute cardiovascular diseases (atrial fibrillation, myocardial infarction, unstable angina, heart failure, and stroke), or peripheral artery disease were excluded. For individuals who participated in ICG, baPWV, and FMD tests, these exams were sequentially performed on the same day. The study protocol was approved by the Ethics Committees of the First Affiliated Hospital of Sun Yat-sen University.

### ICG With the Hemodynamic and Oxygen Transport Management System

The noninvasive hemodynamic parameters were assessed by the HOTMAN system (HEMO SAPIENS Inc. Sedona, Arizona, USA). Studies have validated the device for hemodynamic assessment in hypertensives (28–30). Before ICG test initiation, patients rested in a supine position for at least 5 min. Details of the procedure have been described in previous studies (27, 31). In brief, four dual sensors were placed along the thorax for high frequency, low magnitude measurement current release, and signal detection. Triplicate measurements were performed sequentially within a 10-min test procedure and steady signals were recorded to avoid respiration bias. Blood pressure values were measured on the right upper arm with an electronic blood pressure monitor (HEM-7312, Omron, Kyoto, Japan). Each measurement automatically recorded waveforms signals and calculated hemodynamic parameters, including stroke volume, stroke index (SI), heart rate, cardiac output (CO), cardiac index (CI), stroke systemic vascular resistance index (SSVRI), left stroke work index (LSWI), ejection phase contractility index, inotropic state index (ISI), and thoracic fluid content, which were further defined in Data Supplement and Supplementary Table 1.

### Endothelial Function Assessment

Flow-mediated dilation was measured by high-resolution ultrasonography equipment (UNEXEF18G, UNEX Co., Nagoya, Japan) on the same day following guidelines of the American College of Cardiology (ACC)/American Heart Association (AHA) (32). Measurement details were described in our previous studies (33, 34). In brief, FMD was performed in a temperature-controlled room with participants resting in supine after at least an 8-h fast. A high-resolution transducer was used to visualize the brachial artery longitudinally 5–10 cm above the antecubital crease, and then automatically imaged artery diameter by coupled intellectual software. After acquiring the baseline image for 30 s, the suitable blood pressure cuff, placed around the upper arm, was inflated 50 mmHg greater than the systolic blood pressure (SBP) for 5 min. The artery images were continuously recorded till 1 min after obstruction release. Percentage of FMD [FMD% = (peak diameter—baseline diameter)/baseline diameter] was further used in functional analysis.

### Arterial Stiffness Evaluation

Arterial stiffness was evaluated using baPWV assessed by oscillometry-based technique (BP-203RPE III, Omron, Kyoto, Japan). Measurements were performed in a room with a set temperature. Before measurement initiation, participants were obliged to rest for at least 5 min in a supine position. Four appropriate blood pressure cuffs were placed on bilateral fossa cubitalis and ankles by trained technicians according to the instruction of the manufacturer. Electrocardiograms were synchronously measured by limb leads and thoracic sensors. Bilateral baPWV values were then simultaneously recorded and automatically calculated by the device. BaPWV was defined as the ratio of pulse wave distance divided by the transit time between brachial and posterior tibial arteries. The higher-side baPWV value of each participant was used in further analysis.

### Statistical Analysis

Demographic characteristics and ICG parameters were described among blood pressure categories. Mean and SD, median and interquartile range for continuous variables with or without normal distribution, and percentages for categorical variables were calculated appropriately. Between-group differences were compared using the ANOVA, non-parametric Mann–Whitney *U* test, or the chi-squared tests accordingly. *Post-hoc* comparisons of effects across groups were tested, and Bonferroni correction was used to determine significance. The relationship of FMD, baPWV, and hemodynamic parameters was tested by Pearson's correlation coefficients.

Then, the PCA-based approach (35, 36) was used for dimension reduction of hemodynamics or vascular parameters. The first principal component (PC1) is considered the best representation of variables and utilized to construct SHI and VDI. Upon this approach, dispersed parameters were condensed into a single representative index. In brief, hemodynamic measures were reduced into SHI, whereas demographic and vascular measures were reduced into VDI. Loadings for each PCA-generated index were further illustrated in the Supplementary Figures 1, 2.

A two-tailed *p* < 0.05 was considered statistically significant. All statistical analyses were performed in SPSS version 23 (SPSS Inc., Chicago, Illinois, USA), Origin version 8.5 (OriginLab Corporation, Northampton, MA, USA), and Graphpad Prism version 8 (GraphPad Software Inc., San Diego, California, USA).

## Results

### Population Characteristics

Table 1 shows the clinical characteristics of the study population. A total of 88 participants were included in the final analysis, with a mean (±SD) age of 59.1 (±17.1) years and 56.8% female. SBP ranged from 110 to 187 mmHg. There were no significant differences among blood pressure categories in age, BMI, antihypertensive agents, hypertension duration, and prevalence of diabetes mellitus. The majority of participants were treated by Ca-antagonists. Furthermore, 65 participants with FMD value and 63 with baPWV were involved in functional analysis.

**Table 1:** Clinic characteristics of patients by blood pressure categories.

**Variables**	**SBP**					
	**All (*n* = 88)**	**<130 mmHg**	**130–139 mmHg**	**140–159 mmHg**	**≥160 mmHg**	***p-*** **value**
		**(*n* = 24)**	**(*n* = 20)**	**(*n* = 28)**	**(*n* = 16)**	
Age, years	59.1 (17.1)	57.8 (18.6)	55.7 (17.0)	65.3 (15.6)	54.4 (15.8)	0.1
Female, *n* (%)	50 (57)	11 (46)	14 (70)	16 (57)	9 (56)	0.46
BMI, kg/m^2^	24.8 (3.4)	25.1 (3.5)	24.9 (2.3)	24.9 (3.8)	24.1 (4.0)	0.84
Antihypertensivemedication use, *n* (%)	46 (52)	10 (42)	13 (65)	14 (50)	9 (56)	0.47
RAAS-blockade (ACE and ARB)	28 (32)	6 (25)	10 (50)	5 (18)	7 (44)	0.07
β-blockers	15 (17)	4 (17)	2 (10)	5 (18)	4 (25)	0.7
Ca-antagonists	32 (36)	8 (33)	7 (35)	12 (43)	5 (31)	0.85
Diuretics	7 (8)	3 (13)	1 (5)	1 (4)	2 (13)	0.55
0–5-y duration of hypertension, *n* (%)	57 (65)	18 (75)	11 (55)	17 (61)	11 (69)	0.52
Diabetes mellitus, *n* (%)	13 (15)	5 (21)	1 (5)	4 (14)	3 (19)	0.47
PP, mmHg	57 (13)	50 (9)	53 (11)	61 (10)	66 (15)	<0.001^*^

*Values are expressed as mean (SD), median (interquartile range), or percentage, appropriately. SBP, systolic blood pressure; BMI, body mass index; RAAS, renin-angiotensin-aldosterone system; ACE, angiotensin-converting enzyme; ARB, angiotensin receptor blocker; PP, pulse pressure.*

* *Post-hoc pair-wise comparison showed a significant difference between SBP <130 mm Hg and SBP 140–159 mm Hg; SBP <130 mm Hg and SBP ≥160 mm Hg; SBP 130–139 mm Hg and SBP ≥160 mm Hg*.

### Heterogeneous Hemodynamic and Vascular Function Subsets Exist Regardless of Blood Pressure Stage

To evaluate the systemic hemodynamic parameters variations in different blood pressure categories, a non-invasive ICG technique was performed in volunteers to obtain real-time results. Discrepancies of these parameters among defined groups were summarized in Table 2. SSVRI and LSWI exhibited significant alterations among groups. Especially, in patients who were uncontrolled hypertensives, LSWI tended to progressively increase with elevated SBP degrees, whereas upregulation of SSVRI was not identical. In addition, FMD and baPWV did not exhibit significant variations among groups.

**Table 2:** Comparisons of ICG parameters and vascular function among blood pressure categories.

**Variables**		**SBP**				
	**All (*n* = 88)**	**<130 mmHg**	**130–139 mmHg**	**140–159 mmHg**	**≥160 mmHg**	
		**(*n* = 24)**	**(*n* = 20)**	**(*n* = 28)**	**(*n* = 16)**	***p-*** **value**
**Non-invasive hemodynamics parameters**						
**Blood flow**						
CI, L/min/m^2^	3.3 (1.0)	3.3 (0.9)	2.8 (2.0)	3.1 (1.0)	3.5 (1.1)	0.69
SI, ml/m^2^	48 (14)	51 (13)	41 (18)	48 (14)	48 (14)	0.19
HR, beats/min	68 (13)	66 (13)	72 (11)	66 (17)	71 (12)	0.17
**Resistance**						
SSVRI, dyn·s·cm^−5^·m^2^	178 (59)	129 (43)	182 (78)	166 (63)	192 (102)	<0.001^*^
**Contractility**						
LSWI, g·m/m^2^	66.1 (23.0)	59.3 (16.3)	49.6 (27.1)	68.1 (25.9)	82.5 (42.9)	0.01^†^
EPCI, s^−1^	0.05 (0.03)	0.05 (0.01)	0.04 (0.03)	0.04 (0.01)	0.04 (0.01)	0.44
ISI, s^−2^	0.97 (0.27)	1.03 (0.24)	0.81 (0.44)	0.90 (0.28)	0.97 (0.27)	0.22
**Fluid status**						
TFC Ω^−1^	0.03 (0.00)	0.03 (0.00)	0.03 (0.00)	0.03 (0.00)	0.03 (0.01)	0.33
**Vascular function**						
Arterial compliance	0.9 (0.4)	1.1 (0.4)	0.8 (0.6)	0.8 (0.5)	0.6 (0.4)	0.047
baPWV, cm/s^‡^	1752 (366)	1688 (395)	1699 (292)	1664 (364)	1988 (502)	0.26
FMD, %^§^	5.4 (0.3)	5.6 (3.0)	4.6 (5.0)	4.8 (2.3)	5.4 (2.8)	0.88

*Values are expressed as mean (SD), median (interquartile range), or percentage, appropriately. SBP, systolic blood pressure; CI, cardiac index; SI, stroke index; HR, heart rate; SSVRI, stroke systemic vascular resistance index; LSWI, left stroke work index; EPCI, ejection phase contractility index; ISI, inotropic state index; TFC, thoracis fluid content; Arterial compliance, stroke index/pulse pressure; baPWV, brachial-ankle pulse wave velocity; FMD, flow-mediated dilation*.

* *Post-hoc pair-wise comparison showed a significant difference between SBP <130 mm Hg and SBP 130–139 mm Hg; SBP <130 mm Hg and 140–159 mm Hg; SBP <130 mm Hg and SBP ≥160 mm Hg*.

† *Post-hoc pair-wise comparison showed a significant difference between SBP <130 mm Hg and SBP ≥160 mm Hg*.

‡ *n = 15 for SBP <130 mm Hg; n = 16 for SBP 130–139 mm Hg; n = 19 for SBP 140–159 mm Hg; n = 13 for SBP ≥160 mm Hg*.

§ *n = 16 for SBP <130 mm Hg; n = 17 for SBP 130–139 mm Hg; n = 19 for SBP 140–159 mm Hg; n= 13 for SBP ≥ 160 mm Hg*.

**Figure 1:**
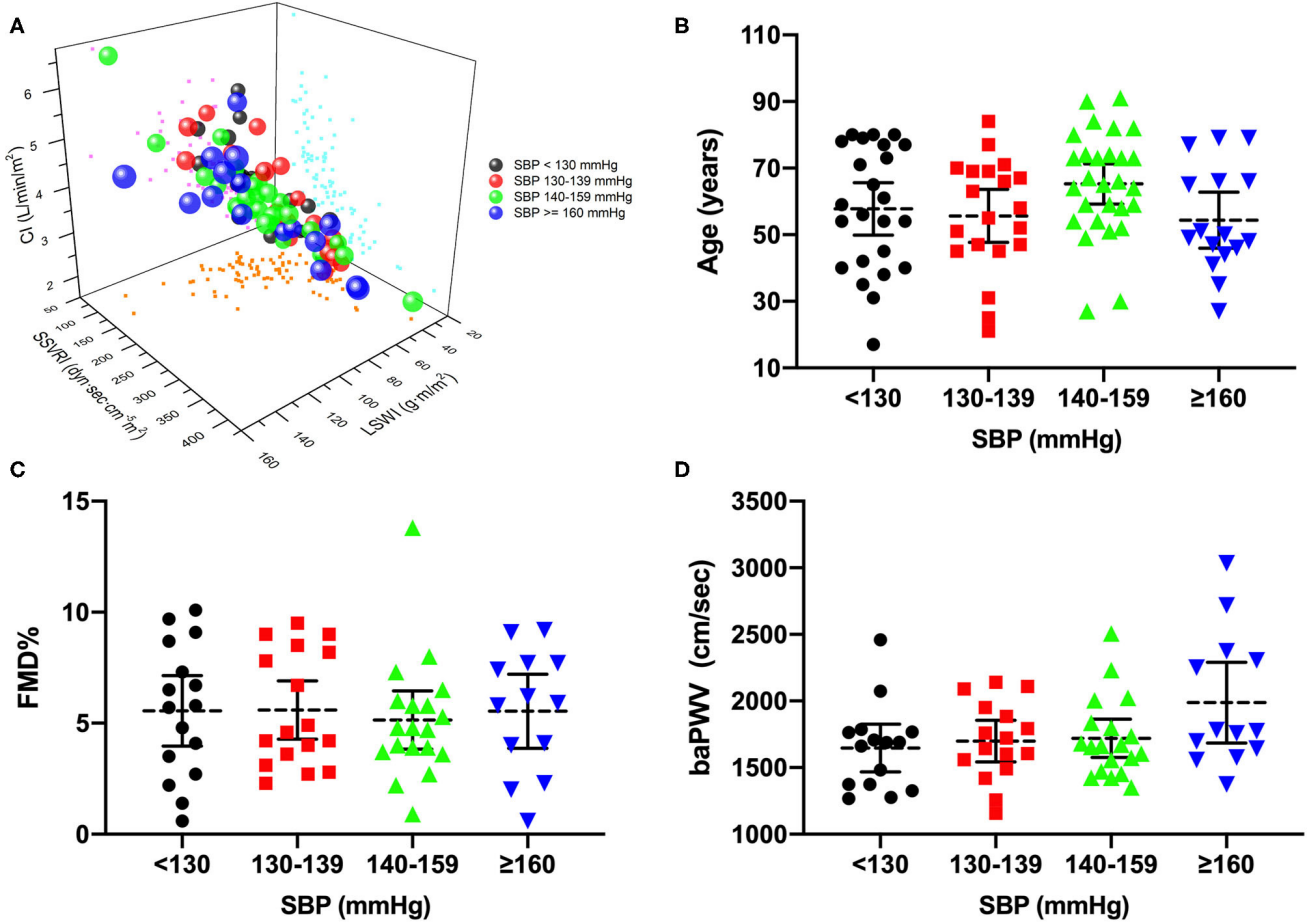
Distribution of hemodynamic components and vascular function of hypertension. **(A)** Three-dimension scatterplot of CI, SSVRI, and LSWI by SBP categories, in which bubble size represents mean arterial pressure level. **(B–D)** Distinct distribution discrepancies of age, FMD, and baPWV in different blood pressure stages. CI, cardiac index; SSVRI, stroke systemic vascular resistance index; LSWI, left stroke work index; SBP, systolic blood pressure; FMD%, flow-mediated dilation percentage of baseline diameter; baPWV, brachial-ankle pulse wave velocity.

However, we discovered distinct alterations of CI, LSWI, and SSVRI within similar degrees of blood pressure elevation (Figure 1A). Similarly, deviations among participants with identical blood pressure stages were found in age, flow-mediated dilation (FMD), and baPWV levels (Figures 1B–D), indicating the additional value of vascular assessment beyond blood pressure levels.

### Hemodynamic Disorders Are Associated With Arterial Endothelial Dysfunction and Increased Arterial Stiffness in Hypertensives

**Figure 2:**
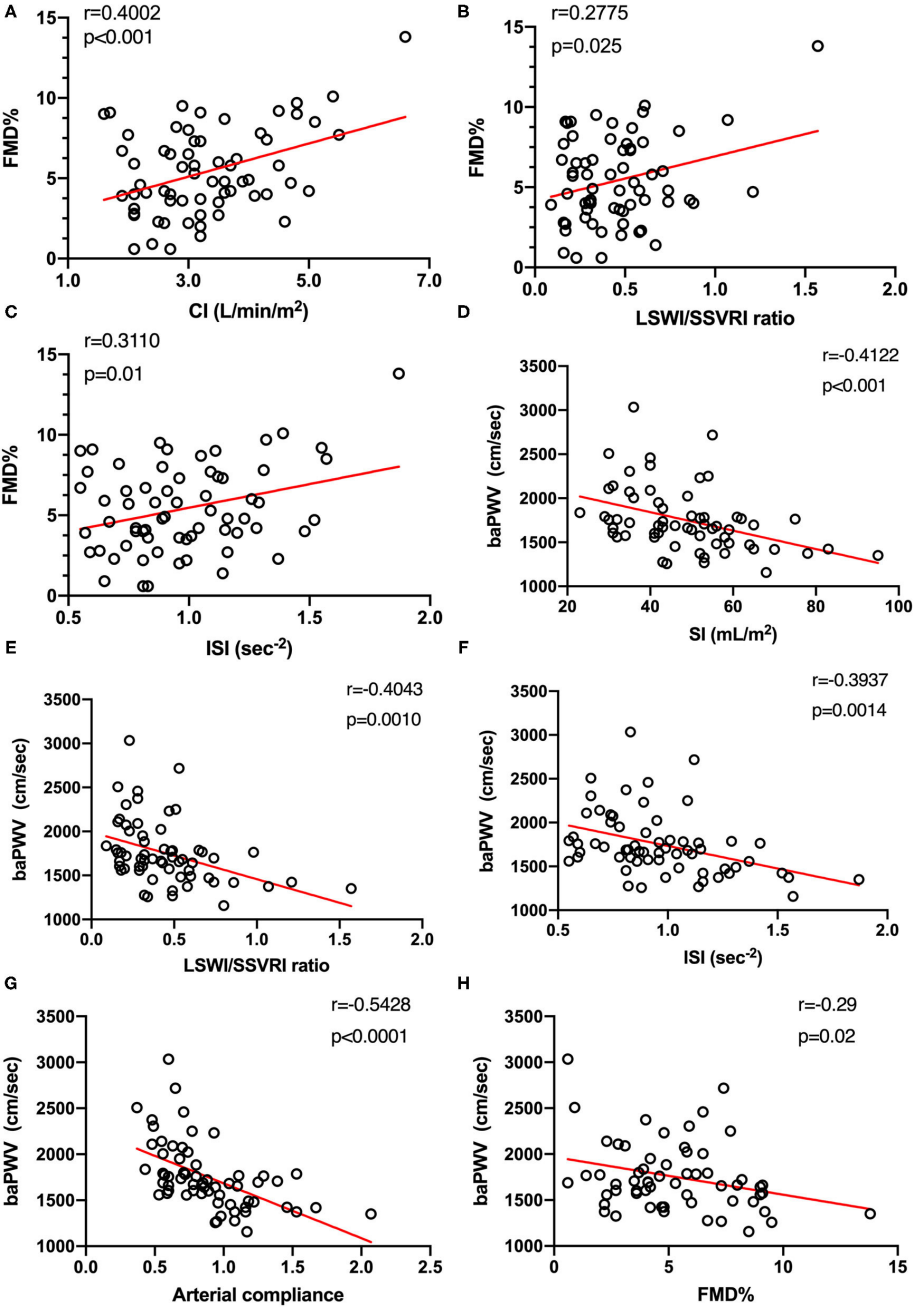
Hemodynamic states are associated with macrovascular endothelial function (*n* = 65) and arterial stiffness (*n* = 63). **(A–C)** Relationship between systemic hemodynamic parameters and FMD was tested using Pearson's correlation coefficients. **(D–F)** Moderate and significant correlations were found between systemic hemodynamic parameters and baPWV. **(G)** Arterial compliance, defined as stroke index divided by pulse pressure, was also used to evaluate arterial stiffness. Both baPWV and arterial compliance explained different aspects of arterial stiffness and were strongly correlated with each other (*n* = 63, *r* = −0.54, *p* < 0.0001). **(H)** A negative moderate (*r* = −0.29) and significant (*p* = 0.02) correlation between arterial stiffness and vascular endothelial function measured by FMD was observed (*n* = 60). FMD%, flow-mediated dilation percentage of baseline diameter; CI, cardiac index; LSWI, left stroke work index; SSVRI, stroke systemic vascular resistance index; ISI, inotropic state index; baPWV, brachial-ankle pulse wave velocity; SI, stroke index.

Figure 2 presents the results of correlation analysis assessing the relationships between FMD, baPWV, and hemodynamic parameters. Our study revealed that CI exhibited a moderate positive correlation with FMD, as well as LSWI/SSVRI ratio (Figures 2A,B). Meanwhile, the inotropy state also exerted a positive influence on endothelial function (Figure 2C). Intriguingly, FMD in different SBP categories did not significantly change (*p* = 0.88), in accordance with the correlation analysis result (*r* = −0.09, *p* = 0.48, Supplementary Figure 3).

In addition, we urged to determine whether systemic hemodynamics was associated with arterial stiffness alteration. Moderate negative correlations between SI, LSWI/SSVRI ratio, ISI, and baPWV were found by Pearson's correlation test (Figures 2D–F). We also observed a moderate negative and significant correlation between arterial compliance and baPWV (*r* = −0.54, *p* < 0.0001, Figure 2G). Despite individual variations, SBP increase was associated with baPWV upregulation (*r* = 0.36, *p* < 0.01, Supplementary Figure 3). Moreover, FMD values showed a moderate negative and significant correlation with baPWV (*r* = −0.29, *p* = 0.02; Figure 2H), consistent with the concept that the combination of arterial endothelial function and arterial stiffness may provide further vascular risk assessment.

### Systemic Hemodynamics Index Decline Is Linked to Hypertension-Related Vascular Impairment

**Figure 3:**
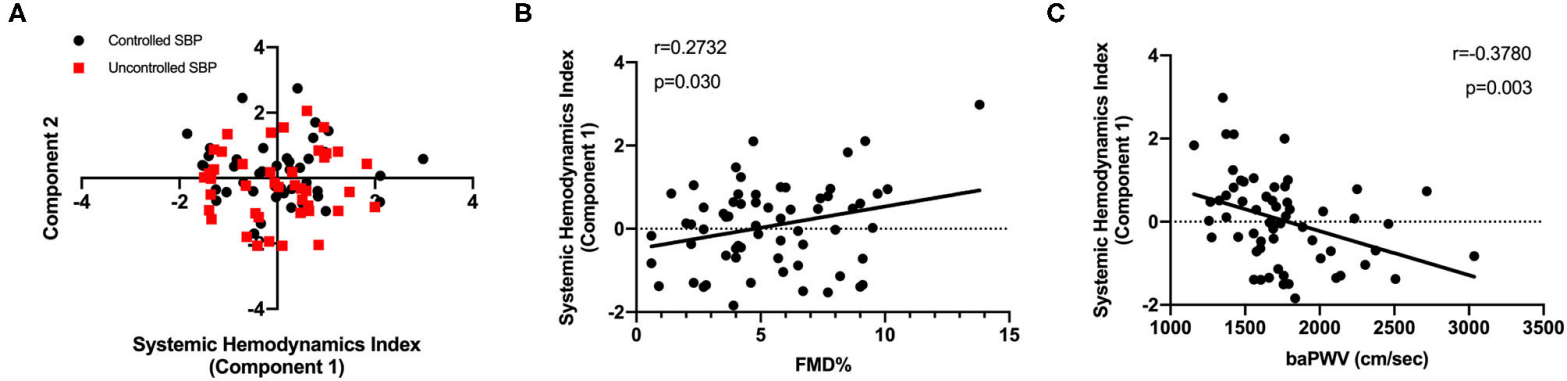
Development of surrogate marker for systemic hemodynamics management. **(A)** A PCA-based method recognized the SHI (principal component 1) as an accounting score. **(B)** A positive and significant correlation of FMD with SHI was displayed (*n* = 65). **(C)** Consistently, the SHI was negatively correlated to baPWV levels (*n* = 63). PCA, principal component analysis; FMD, flow-mediated dilation; baPWV, brachial-ankle pulse wave velocity.

A PCA-based approach was employed to generate a combined SHI as a surrogate marker of hemodynamic states. SHI was comprised of a series of hemodynamic parameters obtained from the ICG test. In this study, PC1, the biggest contributor to hemodynamic states variation (eigenvalue 7.92, Supplementary Figure 1A), was defined as SHI (Figure 3A). The factor loadings (Supplementary Figure 1B) are displayed as the correlation coefficients between PC1 and initial variables. The positive correlation of volume and inotropy parameters with PC1 as well as the negative correlation of arterial resistance with PC1 indicates that high component scores are consistent with a healthier dynamic state. Then, PC1 scores were used to further correlate hemodynamic states changes to hypertension-related vascular function. Significant correlations (Figures 3B,C) were discovered between SHI and baPWV (*r* = −0.38, *p* < 0.01) and FMD (*r* = 0.27, *p* = 0.03).

To integrate factors responsible for hypertensive vascular impairment, we also applied a method based on PCA to construct VDI (including variables of age, hypertension duration, endothelial function, and arterial stiffness). The loadings and composition of VDI are further illustrated in Supplementary Figure 2. The positive correlation of age, hypertension duration, and baPWV with PC1, indicating that increased VDI scores correspond to a progressive hypertension-related vascular impairment.

**Figure 4:**
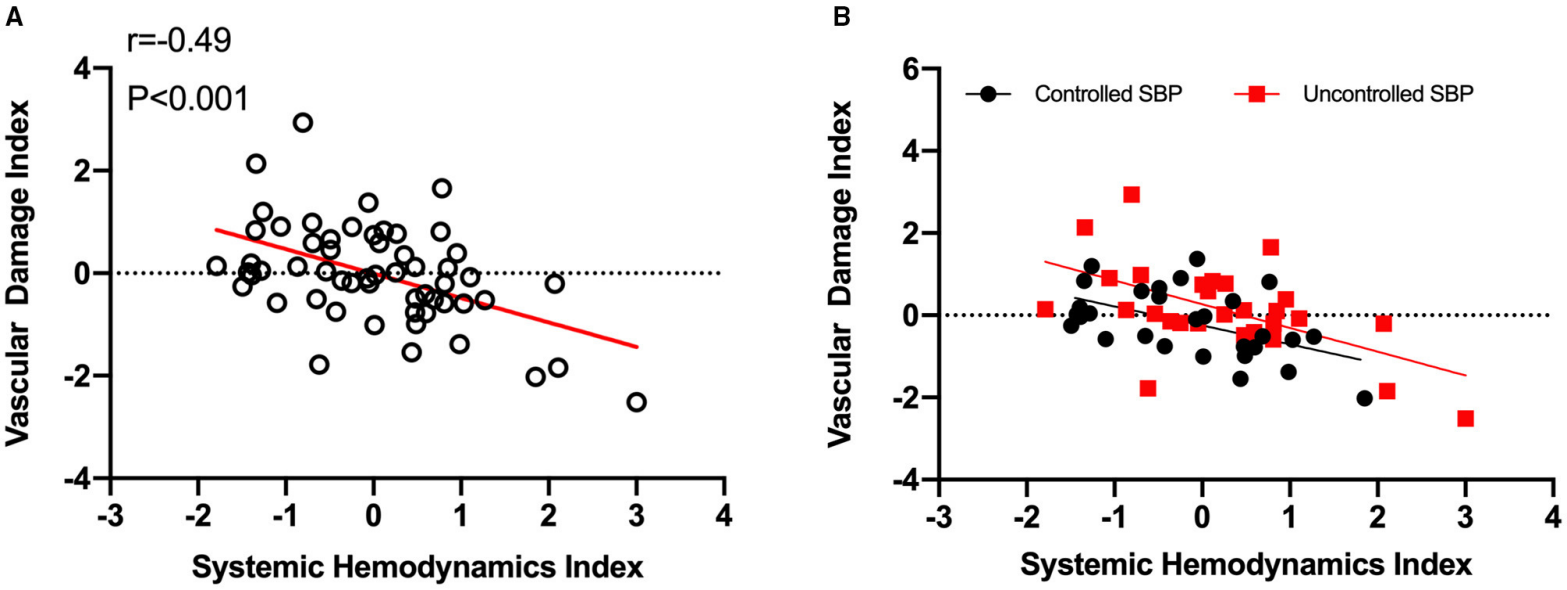
Hypertensive vascular impairment is associated with systemic hemodynamics index decline. **(A)**, Systemic hemodynamics index decrease predicts vascular impairment in hypertensives. The systemic hemodynamics index showed a significantly negative correlation with vascular damage index (*r* = −0.49, *p* < 0.001). **(B)**, Similar tendencies were discovered in both controlled (*n* = 29, *r* = −0.52, *p* < 0.01) and uncontrolled SBP (*n* = 28, *r* = −0.56, *p* < 0.01). SHI, systemic hemodynamics index; VDI, vascular damage index; SBP, systolic blood pressure.

The SHI showed a significantly negative correlation with VDI (*r* = −0.49, *p* < 0.001; Figure 4A). Similar tendencies in controlled and uncontrolled hypertensives (*r* = −0.52, *p* < 0.01; *r* = −0.56, *p* < 0.01, respectively) were also observed (Figure 4B). Accordingly, SHI decline could imply vascular impairment in hypertensives.

## Discussion

This study demonstrates that heterogeneous hemodynamic modulators subsets exist in individuals other than blood pressure values. We discover multiple, significant correlations between hemodynamic parameters and vascular function. To establish a surrogate marker for promising clinical practice, this state-of-art study defines an integrated Systemic Hemodynamic Index (SHI) that inversely correlates with vascular dysfunction and provides information for cardiovascular function assessment tailored for hypertension.

Hypertension correlates with elevated perfusion blood flow, systemic resistance, or both. Our results extend findings of previous researches (31, 37) illustrating that combinations of pulsatile hemodynamic modulators LSWI, SSVRI alteration varies among blood pressure stages or even within identical blood pressure levels. Nonetheless, hypertensives seem to be in conjunction with blood volume elevation, which may explain the beneficial properties of diuretics used in hypertension management (5, 38). More interestingly, treated hypertensives within ideal blood pressure ranges do not necessarily parallel ideal hemodynamics states, which may be partially attributed to empiric prescriptions as reported (39). Taken together, these data highlight the importance of dual-standardized hemodynamic and blood pressure management rather than blood pressure control alone.

Underlying hemodynamic abnormalities in hypertension are responsible for cardiovascular deterioration both in structure and function. Therefore, it suggests that hemodynamics provides more information for vascular risk stratification beyond blood pressure values. Hypertension imposes on the vascular system and damages vascular endothelium and elasticity to various extents. Endothelial dysfunction and arterial stiffness, namely, vascular function impairment, are independent risk factors of cardiovascular events (9, 40, 41). Our study discovers universal correlations between non-invasive hemodynamic parameters and vascular function. Brachial artery FMD, a common approach for endothelial function evaluation (42), is impaired even if SBP is under control. We find a moderate, positive, and significant correlation between FMD and CI indicating that damaged systemic hemodynamics is related to functional abnormalities. In addition, increases in myocardial contractility and parallel decreases in systemic resistance, an amelioration of overall hemodynamics within the reference range, is concomitant with arterial stiffness decline. It is of great significance to assess hypertension-related hemodynamics alteration in vascular risk stratification. Yet, a feasible score designed for direct systemic hemodynamics appraisal has not been reported.

The PCA-based approach allows us to construct comprehensive indices for systemic hemodynamics and vascular damage evaluation, which are subsequently utilized to explore the relationship between changes in hemodynamics and vascular risks on the largest scale. Potential risk predictors, including age, SBP, hypertension duration, number of antihypertensive agents, baPWV, and FMD are chosen as PCA inputs, with the combination of age, hypertension duration, baPWV, and FMD being recognized as VDI, which explains 40.95% of overall variations. Considering the role of blood pressure as hemodynamic formulas parameter and antihypertensive agents' use, SBP is excluded from PCA in this study. Also, other traditional risk factors (e.g., diabetes mellitus, cigarette smoking status, and low-density lipoprotein cholesterol) are not available in this study. Thus, the initial concept of VDI should be cautiously interpreted. In this case, our study provides the first evidence that SHI is negatively correlated to vascular damage. It sheds light on the applicable systemic hemodynamics score for hypertensive vascular risk stratification in clinical practice. Further, SHI may become a potential factor for VDI upgrade, along with other vascular risk confounders, and function as a surrogate marker of cardiovascular outcomes which should be investigated in further large-scale, long-term studies.

There are several limitations to this study. First, due to its cross-sectional design in nature, it is challenging in causality investigation between SHI and vascular dysfunction. Hence, large-scale, prospective, longitudinal studies are required in the future. Second, this study did not report cardiovascular events in the following years. Further researches exploring the correlation between systemic hemodynamics, VDI, and cardiovascular outcomes are warranted. Note that, ICG technique is not applicable in patients with obesity, atrial fibrillation, and severe aortic valve diseases, and shock, which may limit its clinical practice (43). Third, the sample size is rather small. Large cohorts are needed to eliminate confounders and examine the predictive power of SHI for cardiovascular risk stratification in hypertensives.

## Conclusion

In this study, we confirm the heterogeneity of systemic hemodynamics states under blood pressure levels. Of note, the initial SHI and VDI scores may function as feasible tools for vascular damage assessment in clinical practice. Furthermore, the following step is learning to extrapolate potential cardiovascular risk appraisal with solid biomarkers. Future validations in population-based studies are needed.

## Data Availability Statement

The original contributions presented in the study are included in the article/Supplementary Material, further inquiries can be directed to the corresponding author/s.

## Ethics Statement

The studies involving human participants were reviewed and approved by Ethics Committees of the First Affiliated Hospital of Sun Yat-sen University. Written informed consent for participation was not required for this study in accordance with the national legislation and the institutional requirements.

## Author Contributions

JZ, JL, and XZ conducted enrolment of patients and clinical measurements. JZ designed and conducted the statistical analysis, and wrote the original draft of the manuscript. CS and JH helped design the study and review the manuscript. YQ, ZZ, and ZW collected clinical data and provided information on statistical analysis and methodology. BD and QT helped revise the manuscript. SX, WX, and JT participated in the initial study design, wrote and revised the manuscript, and provided guidance in statistical analysis. All the authors have read and agreed to the published version of the manuscript.

## Funding

This study was supported by the National Key Research and Development Program of China (No. 2020YFC2008005), the National Natural Science Foundation of China (Nos. 81671379, 81500205, and 82000466), the Natural Science Foundation of Guangdong Province (No. 2021A1515010914) and the Science and Technology Program of Guangzhou (No. 202002020030).

## Conflict of Interest

The authors declare that the research was conducted in the absence of any commercial or financial relationships that could be construed as a potential conflict of interest.

## Publisher's Note

All claims expressed in this article are solely those of the authors and do not necessarily represent those of their affiliated organizations, or those of the publisher, the editors and the reviewers. Any product that may be evaluated in this article, or claim that may be made by its manufacturer, is not guaranteed or endorsed by the publisher.
