# Supplementary material for: Non-invasive Systemic Hemodynamic Index in Vascular Risk Stratification Tailored for Hypertensives
Source: Front Cardiovasc Med. 2021 Nov 22;8:744349. doi: 10.3389/fcvm.2021.744349 (PMC8645861; doi:10.3389/fcvm.2021.744349)
Supplement: Supplementary file 1 [file Data_Sheet_1.pdf]

Supplementary Material

1 Supplementary Methods

Details of impedance cardiography and calculation of hemodynamic parameters

Impedance cardiography (ICG) technique was used for noninvasive hemodynamics parameters monitor. In brief, the measurement electrical current (high frequency, low magnitude) passes between two pairs of electrodes located on the upper neck and upper abdomen. The thorax acts as an impedance transducer with another four sensing electrodes placed at the levels of neck root and the diaphragm detecting the electrocardiography signals and the high frequency voltage. Change of impedance (ΔZ) is measured by thoracic electron and their rates of their change over time (dZ/dt) is processed. Signal landmarks are extracted to directly measured Thoracic Fluids Conductivity (TFC), Heart Rate (HR), Ventricular Ejection Time (VET). Automatically, algorithms (22) are performed to calculate Ejection Phase Contractility Index (EPCI), Inotropic State Index (ISI), and Stroke Volume (SV). And Body Surface Area (BSA) is determined using the height and weight.

$$SV = VEPT_{gender} \times VET \times EPCI$$

Since vasculature adjusts for every heartbeat, per-beat parameters are used for correct hemodynamics assessment which are calculated as follow:

Stroke Systemic Vascular Resistance Index (SSVRI) = $80 \times (MAP - CVP_{ideal})/SI$

Left Stroke Work Index (LSWI) = $0.0144 \times (MAP - LAP_{ideal}) \times SI$

(MAP, mean arterial pressure; CVP_{ideal}, ideal value of central venous pressure, 4 Torr; LAP_{ideal}, ideal value of left atrial pressure, 7 Torr; SI, stroke index)

2 Supplementary Figures and Tables

Table S1. Noninvasive hemodynamic parameters derived from HOTMAN system.

Parameter	Units	Computation
**Blood flow**		
Stroke Volume	ml	$SV = VEPT_{gender} \times VET \times EPCI$
Stroke Index	ml/m^2	$SI = SV/BSA$
Cardiac Output	L/min	$CO = (SV \times HR)/1000$
Cardiac index	L/min/m^2	$CI = CO/BSA$
Heart rate	beats/min	Derived from R-R intervals of ECG signal
**Resistance**		
Stroke systemic vascular resistance index	dyn·sec·cm^{-5}·m^2	$SSVRI = 80 \times (MAP - CVP_{ideal})/SI$
**Contractility**		
Left stroke work index	g·m/m^2	$LSWI = 0.0144 \times (MAP - LAP_{ideal}) \times SI$
Ejection phase contractility index	sec^{-1}	$EPCI = (\mathrm{dZ/dt})_{\mathrm{max}}/Z_0$
Inotropic state index	sec^{-2}	$ISI = (d^2\mathrm{Z}/dt^2)_{max}/Z_0$
**Fluid status**		
Thoracis fluid content	Ω^{-1}	Derived from ICG waveforms

VEPT, volume of electrically participating tissue; VET, ventricular ejection time; EPCI, ejection phase contractility index; BSA, body surface area; MAP, mean arterial pressure; CVP_{ideal}, ideal value of central venous pressure, 4 Torr; LAP_{ideal}, ideal value of left atrial pressure, 7 Torr.

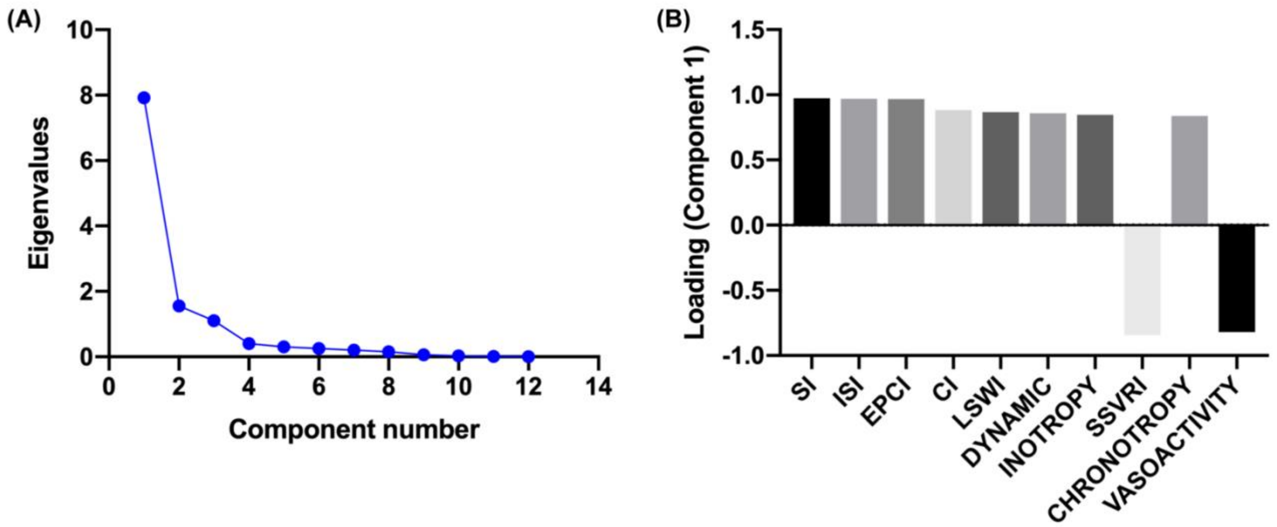

Supplementary Figure 1. PCA approach for systemic hemodynamic index (PC1) construction. **(A)**, Eigenvalues for different components in PCA. **(B)**, The correlation coefficients between PC1 scores and original variables were shown in factors loadings bar.

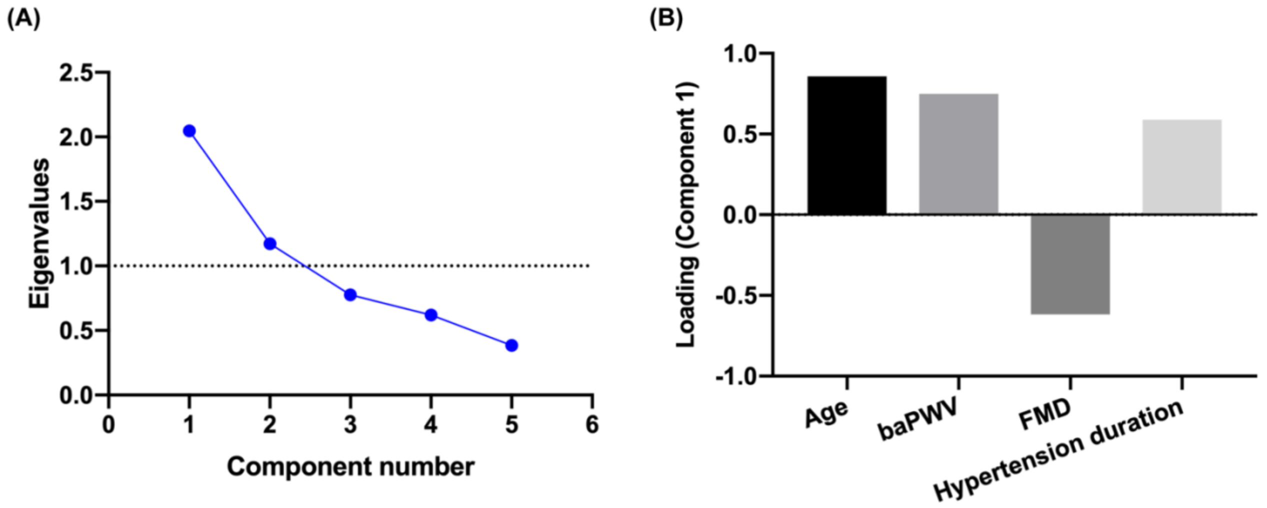

Supplementary Figure 2. PCA approach for vascular damage index (PC1) construction. **(A)**, Eigenvalues for different components in PCA. **(B)**, The correlation coefficients between PC1 scores and original variables were shown in factors loadings bar.

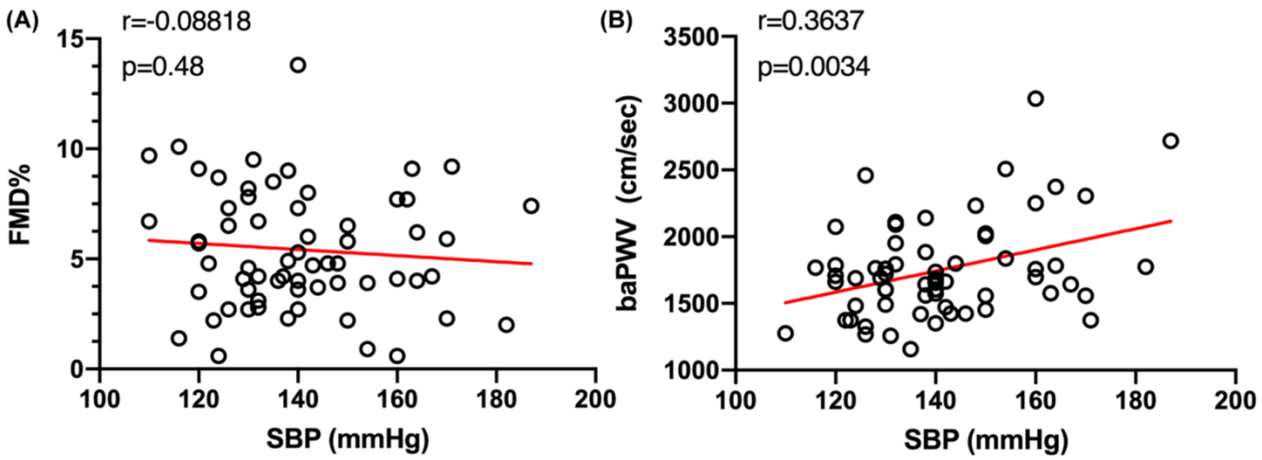

Supplementary Figure 3. Correlation of FMD, baPWV with SBP levels. (A), Relationship between SBP and FMD was tested using Pearson's correlation coefficients (n=65). (B), SBP are correlated with arterial stiffness (n=63). FMD, flow-mediated dilation; SBP, systolic blood pressure; baPWV, brachial-ankle pulse wave velocity.
